# Enteroscrotal Fistula in a Neonate following Incarcerated Inguinal Hernia

**DOI:** 10.21699/jns.v6i3.520

**Published:** 2017-08-10

**Authors:** Prince Raj, Hirendra Birua, Vikash Kumar Prasad

**Affiliations:** Department of Pediatric Surgery, Rajendra Institute of Medical Sciences, Ranchi, India

**DEAR SIR**

Inguinal Hernia is a common surgical problem in children and incarceration is a known complication of inguinal hernia. Management includes early reduction, preferably under sedation with elective herniotomy in the same admission before discharge. Enteroscrotal fecal fistula following incarcerated inguinal hernia is an extremely rare entity, which requires emergency surgical intervention [1-3].

A month old, full-term 2.9 kg male baby, presented with history of passage of stool from the scrotal area for the last 8 days. The child was passing stool normally for the first 18 days of life. On day 19 of life, mother noticed a swelling in the right inguinoscrotal region. Baby started developing abdominal distension and constipation. They sought medical advice in their village on day 23 of life when the child started having fever, irritability, vomiting and progressive increase in the abdominal and inguinoscrotal swelling. On day 25 the child developed scrotal fistula with fecal discharge from it, following which the abdomen settled over next two days. They started feeding the baby and kept him at home for next 6 days due to financial constraints. Currently, he was passing stools exclusively though the scrotal fistula. On examination the child was active, crying, afebrile and with no signs of dehydration. Abdomen was soft, non-tender and bowel sound was present. There was swelling in the right inguino-scrotal region, and on palpation thickened cord with gurgling intestine could be palpated. Perineal examination revealed a fecal fistula on the right of median scrotal raphe with surrounding scrotal and perineal skin excoriation (Fig.1). Blood investigations were within normal limits. Right inguino-scrotal exploration was done till the fistulous opening, as the bowel was adhered to the fistula site. Thick hernia sac with small bowel as content was found. There was perforation in the ileum (20 cm proximal to ileocolic junction) leading to entero-scrotal fistula. Resection of 5 cm of ileum with end to end anastomosis was performed with repair of hernia. Right testis was edematous, though viable. Scrotal wound at fistula site was excised and inguino-scrotal wound was closed in layers. Post operative period was uneventful and the child was discharged on 10th post-operative day. 

**Figure:**
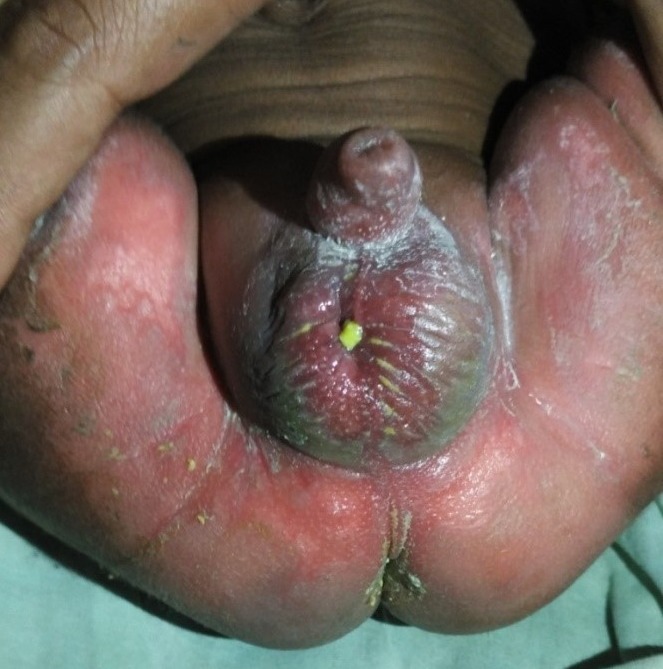
Figure 1: Enteroscrotal fistula

Delay in treatment of inguinal hernia may lead to complication like incarceration and strangulation, with risk of incarceration being as high as 60% in the first 6 months of life [2,3]. Spontaneous bowel necrosis and perforation through the bowel wall of incarcerated inguinal hernia is a rare entity (10 cases reported); these cases presented within the first 2 months of life, with 80% in the first month (including our case) [4,5]. Treatment from quacks, financial constraints, poverty and ignorance on the part of parents in seeking early and proper medical care are the main cause of such complications. It is evident from the fact that all the reported cases are from the developing countries [2-5] including our case. There was one mortality [4]. Ipsilateral testis is also at risk of ischemic injury due to prolonged incarceration, even requiring orchiectomy in two earlier reported cases [4,5]. This further warrant early surgical intervention in case of inguinal hernia. 

To conclude, enteroscrotal fistula is a rare complication of incarcerated inguinal hernia and it reflects poorly on the state of healthcare in our country. The purpose of our reporting was to highlight the need for a prompt and early diagnosis followed by surgical repair of inguinal hernia, particularly in early infancy to prevent such complication.

## Footnotes

**Source of Support:** None

**Conflict of Interest:** None
